# Schwannoma-like uterine leiomyoma with fever of unknown origin and surgical management in a middle-aged woman: A case report

**DOI:** 10.1016/j.radcr.2023.01.094

**Published:** 2023-02-27

**Authors:** Kenji Yorita, Tomotaka Nakagawa, Koki Hirano, Kimiko Nakatani

**Affiliations:** a Department of Diagnostic Pathology, Japanese Red Cross Kochi Hospital, 1-4-63-11 Hadaminamimachi, Kochi-shi, Kochi 780-8562, Japan; b Department of Obstetrics and Gynecology, Japanese Red Cross Kochi Hospital, 1-4-63-11 Hadaminamimachi, Kochi-shi, Kochi 780-8562, Japan; c Department of Radiology, Japanese Red Cross Kochi Hospital, 1-4-63-11 Hadaminamimachi, Kochi-shi, Kochi 780-8562, Japan

**Keywords:** Leiomyoma, Uterus, Schwannoma, Pathology, Differential diagnosis

## Abstract

Herein, we describe a 42-year-old woman with multiple uterine leiomyomas with interesting clinical and histologic findings. She had no medical history, except for uterine myomas, which were diagnosed in her early 30s. She presented with fever and lower abdominal pain, and her symptoms did not respond to antibiotics and antipyretics. The clinical evaluation suggested that degeneration of the largest myoma might be the cause of her symptoms, and pyomyoma was suspected. As she had sustained lower abdominal pain, hysterectomy and bilateral salpingectomy were performed. Histopathological examination confirmed the presence of usual-type uterine leiomyomas without suppurative inflammation. The largest tumor showed a rare morphology with a predominant schwannoma-like growth pattern and infarct-type necrosis. Thus, schwannoma-like leiomyoma was diagnosed. This rare tumor might be one of the manifestations of hereditary leiomyomatosis and renal cell cancer syndrome; however, this patient was unlikely to have that rare syndrome. Herein, the clinical, radiological, and pathologic findings of a schwannoma-like leiomyoma are presented and we have raised the question of whether patients with schwannoma-like uterine leiomyoma are more likely to be associated with hereditary leiomyomatosis and renal cell cancer syndrome than those with usual-type uterine leiomyoma.

## Introduction

Leiomyoma is the most common mesenchymal neoplasm of the uterus. A typical histologic feature of the usual type of uterine leiomyoma is intersecting fascicles of bland spindle cells. Nuclear palisading of the spindle cells and Verocay bodies are characteristic findings of schwannoma. Nuclear palisading of the tumor cells can be seen focally in uterine leiomyoma; however, uterine leiomyomas showing predominantly nuclear palisading have been rarely reported [1], [2], [3]. Herein, we report a rare case of a uterine leiomyoma predominantly showing a schwannoma-like pattern.

Recently, fumarase/fumarate hydratase (FH)-deficient uterine leiomyomas have been reported [4,5]. FH-deficient leiomyoma can be associated with hereditary leiomyomatosis and renal cell cancer (HLRCC) syndrome. Interestingly, a minority of FH-deficient leiomyomas show vague nuclear palisading [4]. Thus, our patient was evaluated for HLRCC syndrome.

## Case report

### Clinical, radiological, and surgical findings

A 42-year-old woman complained of fever and arthralgia. She developed lower abdominal pain 2 days later. Therefore, she went to a nearby hospital. She was gravida 2 para 2 and had not attained menopause. During her previous medical check-up in her early 30s, she had been diagnosed with uterine myomas by ultrasonography. She had a family history of bladder cancer in her paternal grandmother and uterine leiomyomas in her sister and mother. She had no family history of renal cell carcinomas.

Physical examination showed that she had a fever (39°C) and lower abdominal tenderness. No symptoms other than lower abdominal pain were observed. The complete blood test showed a low hemoglobin level (8.6 g/dL) and increased white blood cells (WBC, 10740/µL). Serologic tests showed elevated c-reactive protein (CRP, 4.56 mg/dL). COVID-19 and urinary tract infection were considered unlikely due to negative COVID-19 PCR and urinary tests. Levofloxacin and acetaminophen were administered for several days; however, there was no improvement in her symptoms. Though fever of unknown origin was suspected at first, the patient was referred to our hospital 4 days after the symptom onset with the suspicion of torsion of the uterine myomas.

**Fig. 1:**
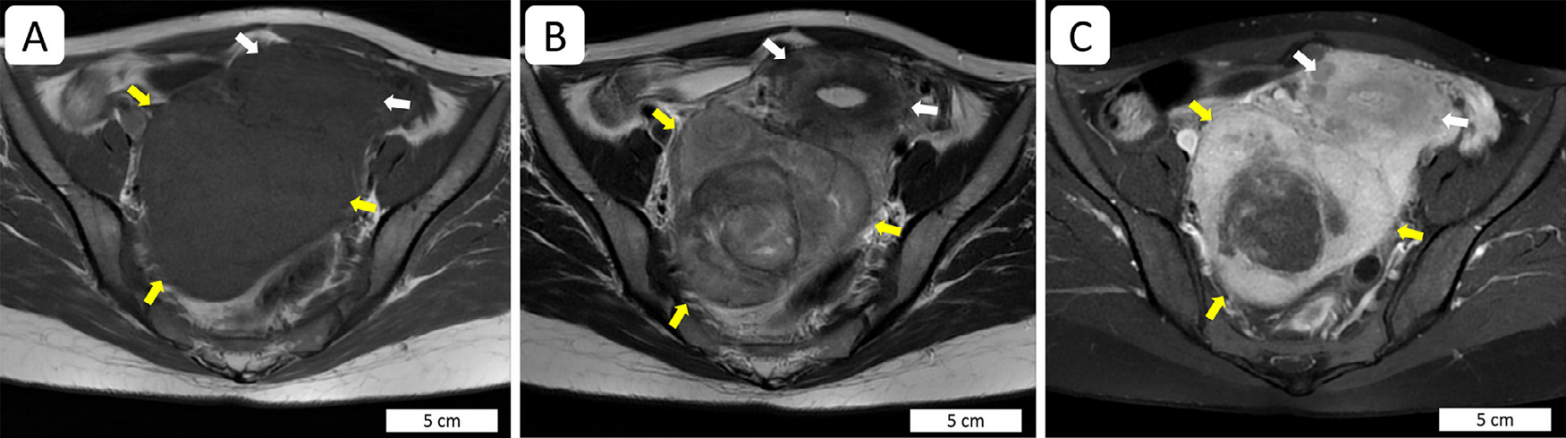
Contrast-enhanced, axial, magnetic resonance images of a schwannoma-like uterine leiomyoma. A schwannoma-like leiomyoma (yellow arrows) is located at the posterior aspect of the uterus (white arrows). The tumor shows low intensity on a T1-weighted image (A) and high intensity on a T2-weighted image (B). The contrast-enhanced image (C) demonstrates a nonenhanced lesion in the tumor. Scale bars are shown in the images.

In our hospital, she had no upper/lower respiratory, gastrointestinal, or urinary symptoms. Her temperature was 37.2°C, and she had lower abdominal pain with tenderness. WBC and CRP were elevated (WBC, 10730/uL; CRP, 7.69 mg/dL), and urine human chorionic gonadotropin was negative. Blood and vaginal cultures were negative. Ultrasonography confirmed multiple uterine masses with the largest mass measuring 12 cm in maximum diameter on the posterior side of the uterus. Contrast magnetic resonance imaging study confirmed multiple masses in the uterine corpus, and most of the masses showed intensity on T1/T2-weighted images similar to that of the myometrium. However, the largest mass on the posterior aspect of the uterus showed higher intensity on T2-weighted images and had a focal nonenhanced area (Fig. 1). Bilateral ovaries demonstrated no abnormalities, and ascites was absent.

A diagnosis of multiple uterine myomas was clinically considered. The degenerated largest myoma appeared to be the cause of fever and lower abdominal pain, with probable superadded infection (pyomyoma) because other infectious etiologies and torsion of the myomas were unlikely. No other differential diagnosis could be suggested. An antibiotic (cefmetazole) was administered, and WBC and CRP decreased after 4 days of medication; however, her lower abdominal pain still remained. Abdominal total hysterectomy and bilateral salpingectomy were performed, given that resection of the myomas was difficult, the patient did not wish to have children in the future, and removal of the fallopian tubes would reduce the incidence of ovarian cancers. Intraoperatively, no abnormal findings except for multiple uterine myomas were observed.

The patient's fever and lower abdominal pain improved after the surgery, and neither the symptoms nor abnormal laboratory data were present 1 month after the surgery. Thus, we considered that the anemia detected before surgery was due to the uterine leiomyomas.

### Pathologic findings

**Fig. 2:**
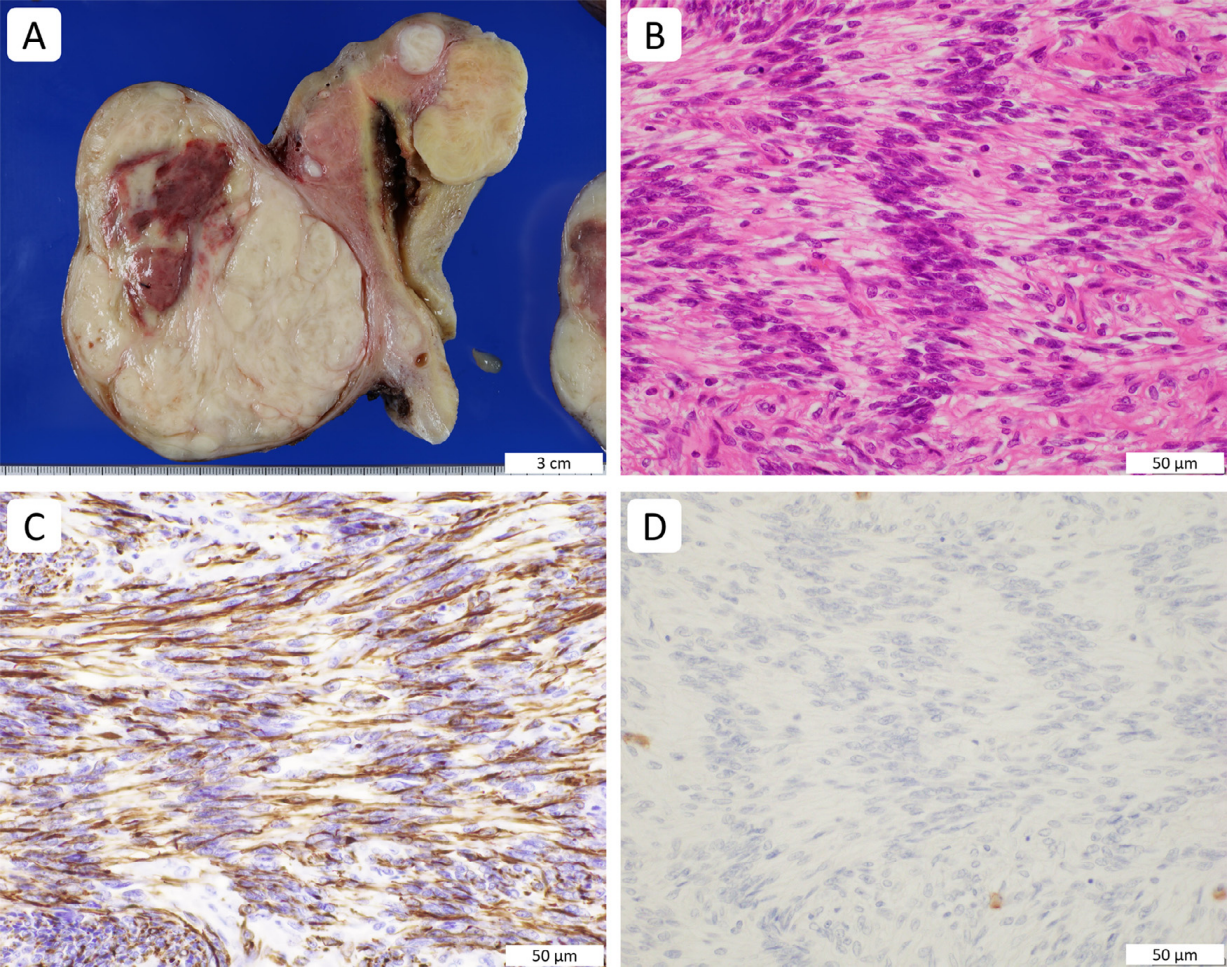
Pathologic images of a schwannoma-like uterine leiomyoma. The sagittal cut surface of the uterus shows a leiomyoma with a red area protruding from the posterior aspect of the uterine corpus (A). Histologically, the subserosal leiomyoma shows nuclear palisading of bland spindle cells and acellular zones forming Verocay bodies (B). Immunohistochemically, the tumor cells are positive for desmin (C) and negative for S100 (D). Scale bars are shown in the images.

Grossly, multiple (approximately 11) demarcated masses were present in the resected uterine corpus, and the largest tumor protruded from the posterior aspect of the uterine corpus. The cut surfaces of the uterine masses were predominantly white to yellowish white; the largest subserosal tumor showed focal hemorrhage and necrosis (Fig. 2A). The maximum cut surface of the largest tumor was processed into histologic specimens, and the smaller masses were also histologically evaluated.

Microscopically, the uterine masses showed interlacing fascicles of bland and uniform spindle cells with eosinophilic cytoplasm. There were rarely any mitoses, and thick-walled vessels and mast cells were seen associated with the tumor cells. Interestingly, nuclear palisading of the spindle cells forming Verocay bodies associated with anuclear zones was observed in 60% of the evaluated area from the largest mass (Fig. 2B), but not in the smaller masses. Hypocellular Antoni B-like areas of schwannoma were not seen. The red areas of the largest tumor showed infarct-type necrosis, which likely corresponded to the non-enhanced region with magnetic resonance imaging. The other masses had no necrosis. No suppurative inflammation was seen in the uterus and fallopian tubes. Neither macroscopic nor microscopic abnormalities were found in the bilateral fallopian tubes.

Histologically, multiple usual-type leiomyomas of the uterine corpus were considered; however, schwannoma and FH-deficient uterine leiomyoma were raised as differential diagnoses in the largest tumor. Immunohistochemically, the spindle cells of the largest tumor were positive for alpha-smooth muscle actin, desmin (Fig. 2C), H-caldesmon, and FH and negative for S100 (Fig. 2D) and SOX10. Ki67 labeling index was 10% (hot spot). Thus, the largest mass was diagnosed as a leiomyoma showing predominantly schwannoma-like growth and infarct-type necrosis.

## Discussion

The patient initially presented with fever of unknown origin. Infarct-type necrosis of the largest uterine leiomyoma appeared to be the cause of fever. Uterine pyomyoma, which is a suppuration resulting from the infarction and infection of a leiomyoma, could be a fatal complication of uterine leiomyomas with a mortality rate of 30% [6]. Thus, clinicians should pay attention to degenerated uterine leiomyomas in patients with fever. Our case showed no pyomyomas.

Herein, schwannoma-like (palisaded) uterine leiomyoma is reported. Our case appeared to be the fourth reported case of such leiomyomas [1], [2], [3]. The 2020 WHO classification described that nuclear palisading may be included in the histologic features of uterine leiomyomas [7]; however, uterine leiomyomas with extensive nuclear palisading and Verocay bodies appear to be rare. The pathogenesis of nuclear palisading in uterine leiomyoma has been unknown. In our case, infarct-type necrosis was seen in the schwannoma-like leiomyoma; however, ischemic change might be unrelated to the growth pattern because the previously published cases of schwannoma-like uterine leiomyoma lacked tumor necrosis [1], [2], [3].

The clinical significance and radiological findings specific to this rare type of uterine leiomyoma have not been reported previously. Also, recurrence was not reported in the previous case [1]. However, a minority of FH-deficient leiomyomas histologically demonstrate vague nuclear palisading [4]. FH-deficient uterine leiomyomas can be associated with HLRCC syndrome [4,5], which is a rare autosomal dominant hereditary tumor syndrome with inactivating germline mutations of the *FH* gene. Immunohistochemistry of FH in uterine leiomyomas is useful for screening patients with suspected HLRCC syndrome [5]. Thus, it may be preferable to evaluate for HLRCC syndrome in patients with schwannoma-like uterine leiomyomas. In our patient, HLRCC was unlikely because of the lack of FH immunoreactivity and family history of RCC.

Nuclear palisading and Verocay bodies are important histologic clues to diagnose schwannoma. Uterine schwannomas are rare and have been reported in the uterine cervix [8,9], not in the uterine corpus. In our case, though the largest tumor contained predominantly schwannoma-like pattern, histologic diagnosis was not difficult because the typical features of uterine leiomyoma were also seen in the largest tumor, and immunohistochemical evaluation confirmed smooth muscle differentiation and lack of neurogenic differentiation in the largest tumor.

In conclusion, we have reported a case of a woman with a schwannoma-like uterine leiomyoma. The patient did not have HLRCC syndrome. Although the clinical significance of this rare uterine tumor has not yet been reported, this tumor might be related to the HLRCC syndrome. Further studies are required to examine whether patients with schwannoma-like uterine leiomyoma are more likely to be associated with HLRCC syndrome than those with usual-type uterine leiomyoma.

## Patient consent

Written informed consent was obtained from the patient for the publication of this case report.
